# Lentiviral-Induced Spinal Cord Gliomas in Rat Model

**DOI:** 10.3390/ijms222312943

**Published:** 2021-11-30

**Authors:** Purva P. Nagarajan, Muhibullah S. Tora, Stewart G. Neill, Thais Federici, Pavlos Texakalidis, Anthony Donsante, Peter Canoll, Kecheng Lei, Nicholas M. Boulis

**Affiliations:** 1 Department of Neurosurgery, Emory University School of Medicine, Atlanta, GA 30322, USA; purva.parvathy.nagarajan@emory.edu (P.P.N.); mohib.tora@emory.edu (M.S.T.); thais.buchman@emory.edu (T.F.); Pavlos.texakalidis@emory.edu (P.T.); anthony.donsante@emory.edu (A.D.); 2 Department of Biomedical Engineering, Georgia Institute of Technology, Atlanta, GA 30332, USA; 3 Department of Pathology and Laboratory Medicine, Emory University School of Medicine, Atlanta, GA 30322, USA; sgneill@emory.edu; 4 Department of Pathology and Cell Biology, Columbia University, New York, NY 10032, USA; pc561@cumc.columbia.edu

**Keywords:** spinal cord glioma, lentivirus, PDGF-B, P53, HRAS, rat model

## Abstract

Intramedullary spinal cord tumors are a rare and understudied cancer with poor treatment options and prognosis. Our prior study used a combination of PDGF-B, HRAS, and p53 knockdown to induce the development of high-grade glioma in the spinal cords of minipigs. In this study, we evaluate the ability of each vector alone and combinations of vectors to produce high-grade spinal cord gliomas. Eight groups of rats (*n* = 8/group) underwent thoracolumbar laminectomy and injection of lentiviral vector in the lateral white matter of the spinal cord. Each group received a different combination of lentiviral vectors expressing PDGF-B, a constitutively active HRAS mutant, or shRNA targeting p53, or a control vector. All animals were monitored once per week for clinical deficits for 98 days. Tissues were harvested and analyzed using hematoxylin and eosin (H&E) and immunohistochemical (IHC) staining. Rats injected with PDGF-B+HRAS+sh-p53 (triple cocktail) exhibited statistically significant declines in all behavioral measures (Basso Beattie Bresnahan scoring, Tarlov scoring, weight, and survival rate) over time when compared to the control. Histologically, all groups except the control and those injected with sh-p53 displayed the development of tumors at the injection site, although there were differences in the rate of tumor growth and the histopathological features of the lesions between groups. Examination of immunohistochemistry revealed rats receiving triple cocktail displayed the largest and most significant increase in the Ki67 proliferation index and GFAP positivity than any other group. PDGF-B+HRAS also displayed a significant increase in the Ki67 proliferation index. Rats receiving PDGF-B alone and PDGF-B+ sh-p53 displayed more a significant increase in SOX2-positive staining than in any other group. We found that different vector combinations produced differing high-grade glioma models in rodents. The combination of all three vectors produced a model of high-grade glioma more efficiently and aggressively with respect to behavioral, physiological, and histological characteristics than the rest of the vector combinations. Thus, the present rat model of spinal cord glioma may potentially be used to evaluate therapeutic strategies in the future.

## 1. Introduction

Spinal cord glioma (SCG) is a rare type of cancer, making up approximately 2–6% of all cancers of the central nervous system [1,2]. High-grade glioma of the spinal cord has universally poor outcomes, leading either to death from, or long-term suffering due to, ascending paralysis [3]. Symptoms of this disease often include a loss of sensation as well as pain. Many institutional case series report that patients with high-grade spinal cord astrocytoma do not survive more than 2 years [4]. High-grade gliomas can be classified into many histological subtypes based on their cellular origins such as glioblastoma, oligodendroglioma, astrocytoma, and ependymoma. Histologic and molecular characterization is the current standard for the classification and grading of glioma in patients. Recently, Broggi et al. demonstrated that serine- and arginine-rich splicing factor 1 (SRSF1) immunohistochemical expression may be a diagnostic marker of astrocytomas and oligodendrogliomas. The absence of this expression may also help confirm ependymoma and pilocytic astrocytoma diagnosis [5]. Similarly, serum extracellular vesicle-derived circular RNAs circSMARCA5 and circHIPK3 can be used as biomarkers for GBM in conjunction with other known preoperative diagnostic markers [6]. Current treatments for high-grade glioma include resection of the tumor, chemotherapy, radiation, and convection-enhanced delivery (CED) of oncolytic viruses or chemotherapy [7]. Since glioma presents with individual differences in genomic and phenotypic makeup, as well as heterogeneity within individual masses, treatment options need to target a variety of cell types and growth patterns, while minimizing damage to healthy tissue [8]. Image complete resection is rare, due to the eloquence of surrounding tissue [9]. Moreover, there is rarely a dissection plane present to enable resection [10]. There has been little improvement in treatment for this debilitating disease in the last few decades, partly due to the severity of the disease itself.

Many of the existing animal models of SCG have significant limitations [11]. The most common models of glioma are based on transgenic mice or the transplantation of allogenic or xenogenic tumor cell lines. Xenografts require immunosuppression and display non-invasive growth patterns. Moreover, transgenic models have gene alteration capabilities and are often highly variable in the location of lesion formation, phenotype, latency, and penetrance [4,12]. Due to the limitations of these models, a more reliable and translatable model of spinal cord glioma is needed to better test emerging and existing therapies for the disease.

A viral vector model can be used in a large immunocompetent animal, providing the ability to precisely control the tumor location, the time span of formation, and cellular origin [4]. Previously, we have generated intermedullary spinal cord glioma as well as malignant brain glioma in rats using a retroviral vector expressing platelet-derived growth factor B (PDGF-B) [12,13]. The former study presented the first testimony of the innate tumorigenic potential of the adult spinal cord. Furthermore, the transforming qualities of PDGF-B overexpression in the glial cells of the spinal cord can be enhanced with the use of a lentivirus, by producing a more penetrant disease model [14,15]. Genomic analysis of human high-grade gliomas has shown that genetic alterations occur in multiple pathways. High-grade gliomas are characterized by mutations in the receptor tyrosine kinase (RTK)/rat sarcoma viral oncogene homolog (RAS)/ phosphatidylinositol 3′-kinase (PI3K) signaling pathway, which lead to increased cell proliferation [16]. Prior studies have also shown that in almost 90% of adult human gliomas, RTK/RAS/PI3K signaling is altered [17]. Alterations in the p14ARF/CDKN2A and TP53 signaling pathway, which is associated with a loss of apoptotic control, are also observed in human glioma [18]. RB/INK4a signaling is affiliated with cell cycle progression, specifically at the checkpoint of the G1/S boundary [19]. These pathways have inspired numerous glioma models based on the retroviral expression of PDGF-B, HRAS-G12V, AKT, and IDH1-R132H [20].

Concerns about the ability of existing models to faithfully replicate human gliomas led us to produce the first lentiviral-vector-induced models of high-grade spinal cord glioma in rats and pigs [12,21]. Recently, we reported that the virus cocktail (PDGF-B, HRAS-G12V, and shRNA-p53) induces glioma in the minipig spinal cord [21]. Because these oncogenes have been shown to produce glioma in the brain, it is essential to determine the effect of these vectors, in different combinations, in spinal cord glioma formation. Our present study aims to determine the efficacy of each lentiviral vector on its own, as well as two-vector combinations, against the original three-vector cocktail and the control in producing a high-grade spinal cord glioma model in rats. Moreover, we have characterized the behavior and histopathology of our model. The present manuscript provides qualitative and quantitative confirmation of the production of high-grade glioma in the spinal cord using oncogenic lentivectors. We believe that this model will provide a means for preclinical testing of treatment options.

## 2. Results

### 2.1. Animals Injected with HRAS and Triple Cocktail Exhibit Aggressive Motor Deficits

**Figure 1:**
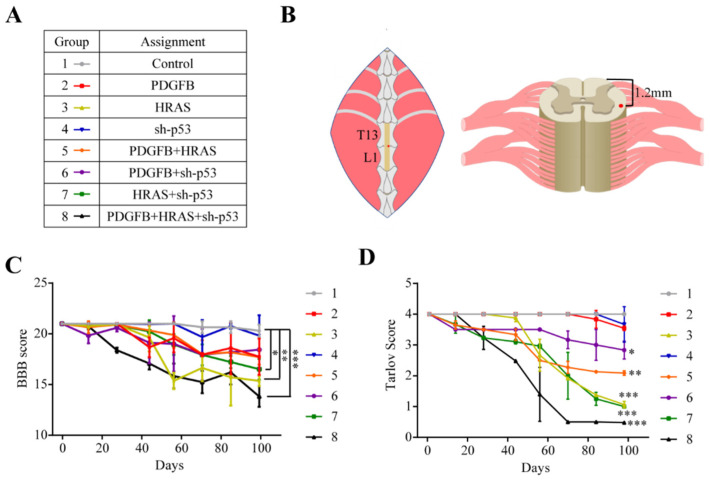
Experimental groups, surgery, and behavioral outcomes. (**A**) Vector assignments for experimental groups 1–8. (**B**) Surgical approach and injection target. Exposure of thoracolumbar spinal cord with location of injection target in the region of the spinal cord coinciding with the thoracolumbar junction of the vertebrae. Cross-section of spinal cord showing depth and location of injection target (red dot). (**C**) BBB progression over time. (**D**) Tarlov progression over time. Results are shown as means ± SEM; *n* = 8 each group; * *p* < 0.05; ** *p* < 0.01; *** *p* < 0.001, one-way ANOVA.

Basso Beattie Bresnahan (BBB) Scoring is a 21-point locomotor recovery scaling system that is used to assess behavioral consequences of spinal cord injury in rodents [22]. Rats injected with the HRAS vector alone (group 3) exhibited statistically significant declines in BBB scoring over time when compared to the control. The same effect was seen in rats injected with the triple cocktail (group 8). All other groups did not show significantly different scoring declines over time compared to the control (Figure 1C).

Similar to the BBB test, rats in groups injected with HRAS alone, PDGF-B+HRAS, PDGF-B+sh-p53, HRAS+sh-p53, and triple cocktail (groups 3, 5, 6, 7, and 8) exhibited statistically significant declines in Tarlov scoring over time when compared to the control. Groups injected with PDGF-B alone (group 2) and sh-p53 alone (group 4) did not display significantly different declines in Tarlov scoring when compared to the control (Figure 1D). Upon observation of gross anatomy, all animals exhibiting severe declines in motor function had macroscopically visible lesions. In summary, rats injected with HRAS alone and the triple cocktail exhibited the most aggressive phenotype with respect to BBB and Tarlov scoring.

### 2.2. Animals Injected with Triple Cocktail Show Significant Decline in Weight and Survival Rate

**Figure 2:**
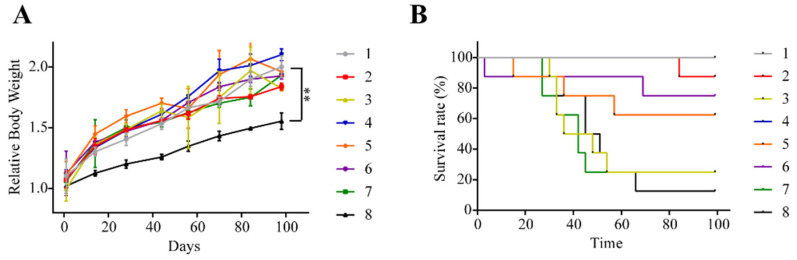
Body weight changes and survival rate. (**A**) Rats injected with triple cocktail (group 8) showed statistically significant declines in relative body weight over time when compared to control. Results are shown as means ± SEM; *n* = 8 each group; ** *p* < 0.01, one-way ANOVA. (**B**) The survival rate for all groups.

Rats injected with the triple cocktail displayed a significant decrease in relative body weight when compared to control. This effect may be due to the onset of motor deficits hindering access to the food container. It could also be attributed to nausea or a loss of appetite from lesion development (Figure 2A). Groups injected with HRAS (group 3), HRAS+sh-p53 (group 7), and the triple cocktail (group 8) exhibited significant declines in the survival rate when compared to all other groups (Figure 2B). This may be attributed to longer tumor progression timelines for the other vector combinations. In summary, animals injected with the three-vector combination had the worst outcome functionally, resulting in a significant decline in both bodyweight and the survival rate.

### 2.3. Histopathological Confirmation of Glioma Characteristic Differences between Groups

**Figure 3:**
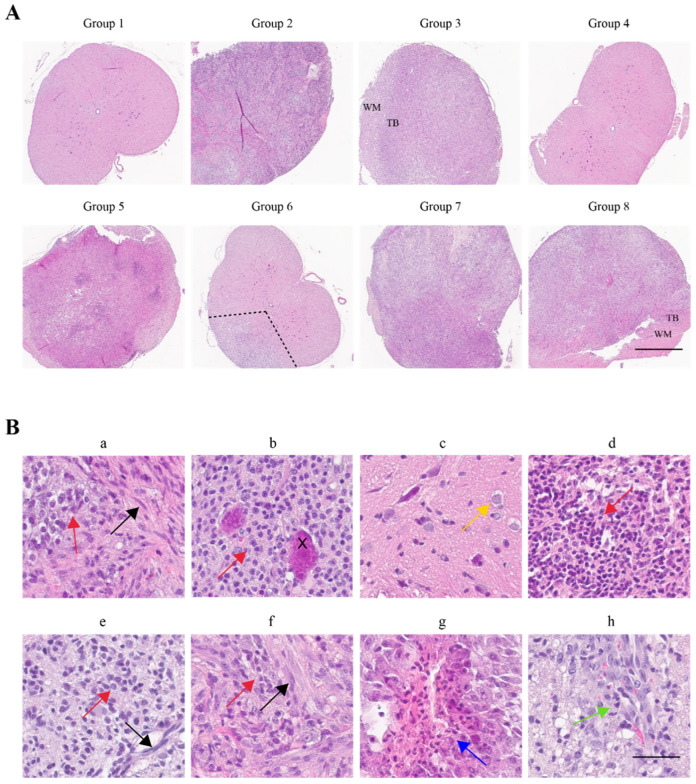
Histopathologic characterization of spinal cord lesions. (**A**) H&E staining at low magnification in different groups. Histopathologic features were investigated at low magnification, observing invasion along the tumor border (TB) into surrounding white matter (WM) (Groups 3, 8). Tumor formation was observed in Group 6, infiltrating only around 25% of the spinal cord cross section (dotted line). Scale Bar = 1 mm. (**B**) H&E staining at high magnification in different groups. Histopathologic features were investigated at high magnification, noting high cellularity (red arrows, panels **a**,**b**,**d**–**f**) with fibrillary astrocytic morphology (black arrows, panels **a**,**e**,**f**), regions of necrosis (blue arrow, panel **g**), and microvascular proliferation (green arrow, panel **h**). All groups except those injected with sh-p53 alone (group 4) and the control (group 1) displayed these features. In addition, background parenchyma was visible in regions of the tumor, here depicting a motor neuron of the ventral horn (black “X”, panel **b**). The classic “fried-egg” cell structure seen in oligodendroglioma was also found in groups injected with PDGF-B alone and PDGF-B+sh-p53 (yellow arrow, panel **c**). Scale Bar = 50 μm.

H&E staining is used to evaluate hyper-cellularity, necrosis, invasion along the tumor border, tissue expansion, and pronounced tumor border for glial sarcoma-like lesions at low magnification. Histopathologic features identified at low magnification include the invasion along the tumor border (TB) into the surrounding white matter (WM) in groups injected with HRAS alone and the triple cocktail (groups 3 and 8, respectively). Initial stages of tumor formation were observed in the group injected with PDGF-B+sh-p53 (group 6), infiltrating only around 25% of the spinal cord cross section, suggesting a slower lesion growth rate (Figure 3A). Histopathologic features identified at high magnification included high cellularity (red arrows), fibrillary astrocytic morphology (black arrows), and regions of pseudo-palisading necrosis (blue arrow), which were all displayed in all groups except sh-p53 alone and the control (groups 4 and 1, respectively) (Figure 3B). Microvascular proliferation (MVP) is a marker of poor prognosis for patients with GBM [23]. This feature was demonstrated in all groups except those injected with sh-p53 alone and the control (groups 4 and 1, respectively) (Figure 3(Bh), green arrow). Because MVP is often seen in conjunction with necrosis, individuals with aggressive glioma that displayed necrosis also displayed high cellularity, fibrillary astrocytic morphology, and MVP in most cases. Additionally, entrapped neurons are visibly interspersed throughout and at the periphery of the tumors, here depicting a motor neuron of the ventral horn surrounded by high cellularity (Figure 3(Bb), black “X”). Depictions of the classic “fried-egg” cell structure in groups injected with PDGF-B alone and PDGF-B+sh-p53 suggests that oligodendroglioma-like lesion formation occurs with these vector combinations (Figure 3(Bc), yellow arrow).

Evaluation of these characteristics resulted in the assignment of individuals as aggressive (resemblance to GBM or glial sarcoma), infiltrative, or less-aggressive tumor formations. Groups categorized from most to least aggressive per these observations are as follows: The triple cocktail group contained the most-aggressive tumor formation cases. HRAS+sh-p53 (group 7) and PDGF-B alone (group 2) contained approximately equal cases of aggressive lesions and infiltrative glioma. HRAS alone (group 3) and PDGF-B+HRAS (group 5) groups consisted of a mixture of aggressive, infiltrative, and less-aggressive cases. PDGF-B+sh-p53 (group 6) contained a mixture of aggressive, less-aggressive, and no-lesion cases, with most cases being less-aggressive. Finally, the sh-p53 and control groups (group 4) contained no lesion cases.

### 2.4. Immunohistochemical Confirmation of Glioma Marker Differences between Groups

**Figure 4:**
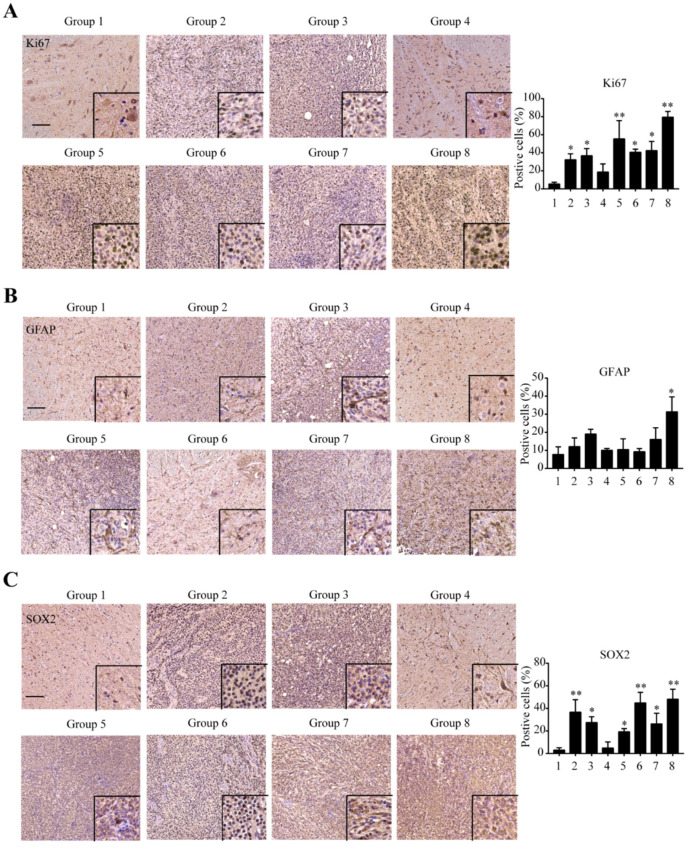
Immunohistochemical characterization of spinal cord lesions. Standard fixed, paraffin-embedded staining protocols with citrate or tris-mediated antigen retrieval were applied to 8um-thick serial sections. (**A**) IHC staining for Ki-67, a marker of proliferation, in different groups All groups except those injected with sh-p53 alone (group 4) displayed a significant increase in Ki-67 proliferative index compared to control. Rats receiving PDGF-B+HRAS (group 5) and triple cocktail (group 8) displayed largest and most significant increase in Ki67 proliferation index than any other group (Figure 4A). (**B**) IHC staining for GFAP, a marker of astrocytic phenotype, in different groups. Significant increase in positive staining for GFAP only occurred in rats injected with the triple cocktail (group 8). (**C**) IHC staining for SOX2, a pan glioma marker, in different groups. All groups except those injected with sh-p53 alone (group 4) displayed significant increase in SOX2-positive staining compared to control. Rats receiving PDGF-B alone, PDGF-B+sh-p53, and triple cocktail (groups 2, 6, and 8) displayed more significant increase in SOX2-positive staining than in any other group. Scale Bar = 100 μm. Data are means ±SEM (* *p* < 0.05; ** *p* < 0.01, one-way ANOVA).

To confirm that our vectors were expressed in our target tissue, we stained for PDGF-B, HRAS, and p53. As expected, PDGF-B-positive staining was displayed in the four groups injected with PDGF-B (groups 2, 5, 6, 8). HRAS positivity was seen in the four groups injected with HRAS (groups 3, 5, 7, 8). P53 positive staining was displayed in the groups not injected with our p53 knockdown (groups 1, 2, 3, 5). These staining patterns confirm that our vectors were delivered to the target tissue and that they were expressed sufficiently (Figure S1). To further characterize the tumors, sections were stained for Ki-67 for the proliferative index [17]. All groups except those injected with sh-p53 alone (group 4) displayed significantly more positive staining for Ki-67 than in the control. Rats receiving PDGF-B+HRAS (group 5) and the triple cocktail (group 8) displayed significance with lower p-values for positive staining than in any other group (Figure 4A). Cell proliferation within the hypercellular regions of these groups confirms the invasive nature of the lesion. To gain a better understanding of the severity and cellular makeup of the tumors, adjacent sections were stained for GFAP and SOX2. GFAP positivity is used for the confirmation of the astrocytic phenotype. Significant positive staining for GFAP only occurred in rats injected with the triple cocktail (group 8) (Figure 4B). SOX2 is a pan glioma marker that has been shown to be the most pervasively expressed gene in high-grade gliomas [24]. Positive staining coincides with hypercellular areas with high vessel density. SOX2 stained significantly positive in rats injected with PDGF-B alone, HRAS alone, PDGF-B+HRAS, PDGF-B+sh-p53, HRAS+sh-p53, and the triple cocktail (groups 2, 3, 5, 6, 7, and 8, respectively) (Figure 4C). Above all, our triple cocktail model displayed staining that indicates both astrocytic and high-grade glioma characteristics that resemble human GBM. Stains for Olig2 were performed to investigate additional differences in our lesions between groups. Olig2-positive staining is a marker of glioma of oligodendroglial origin [25]. According to our previous work, administration of the triple cocktail model produces a lesion that stains highly positive for Olig2 in minipig spinal cords [21]. IHC staining for Olig2 in our rat spinal cord models displayed increased positive staining in groups injected with HRAS alone, PDGF-B+HRAS, PDGF-B+sh-p53, HRAS+sh-p53, and the triple cocktail (groups 3, 5, 6, 7, 8) (Figure S2A). These staining patterns indicate that certain vector combinations induce lesions of oligodendroglial lineage while other combinations do not.

Given these staining patterns, different vector combinations appear to give rise to different gliomas, with characteristics in H&E and markers in IHC differing between groups.

## 3. Discussion

In the present study, the combination of the three lentiviral vectors expressing PDGF-B, HRAS-G12V, and shRNAp53 produced the most-aggressive tumors, behaviorally, grossly, and histologically. Interestingly, shRNA targeting p53 alone failed to induce glioma formation in a 98-day period.

SCG in humans is relentless. Five-year survival rates are in the range of 40–60% when treated with radiation therapy and resection [26]. New therapies emerge every day; thus, more robust models of SCG are needed in order to test the true efficacy of new therapies without the hinderance of immunosuppression and externally produced tumor cells. The injection of the lentiviral three-vector cocktail into the spinal cord of rats in the present study offers a robust model of highly aggressive SCG, while other conditions of lentiviral vectors, alone or in combinations, can provide less aggressive SCG with distinct genetic and histopathological features.

Only a handful of studies have attempted to create a spinal cord glioma model, the first of which was in canines in 1984 [27]. To produce this model, the investigators xenografted tumor cells, injecting them into the thoracic region of the spinal cord in 2–3 sites per animal. Overall, 80% of animals developed tumors and showed deficits within 2 weeks of surgery. Although this model is sufficient in mimicking the gross anatomy of SCG, it lacks immunological data. Moreover, the model is cumbersome for preclinical testing, as it requires housing for a large number of subject animals, long surgical time, and a high cost per animal. Mavinkurve et al. developed a rabbit model of intermedullary SCG using the VX2 squamous cell carcinoma cell line. This model produced more consistent and predictable symptoms and reproduced the histopathologic and radiologic characteristics of human glioma. A total of 100% of animals developed paraparesis approximately 17 days post injection. Despite reproducing features of glioma, the study used a squamous cell carcinoma cell line of epithelial origin, while most human SCGs are of glial origin [28]. This difference calls into question its viability to serve as a model of SCG. The transgenic model reported by Hitoshi et al. avoids this issue but relies on a longer timeline for formation with variable locations of formation and progression [4]. The use of lentivirus provides greater flexibility because lentiviral vectors are able to integrate into the genome, increasing the possibility of tumor formation through the maintenance of expression in daughter cells in the proliferative environment. Additionally, second- and third-generation replication-deficient lentiviruses allow for a large insert size, low immune feedback, and broad vector tropism with a VSV-G pseudotype [21]. The lentiviral vector is often used in gliomagenesis because of its ability to infect both dividing and postmitotic cells [29,30]. The lentivirus has the ability to target a wide variety of cells, including astrocytes, oligodendrocytes, endothelial cells, and microglia [20,31,32]. Moreover, it was interesting that lentivirus can also efficiently deliver into CD4 T cells [33], CD8 T cells [34], and CD20 B cells [35]. The use of lentiviral vectors in the present study permits the development of a histologically and anatomically invasive tumor in the spinal cord.

Spinal cord glioma and their cerebral counterparts both fall under the category of glioblastoma. Lee et al. published findings on genetic differences between spinal versus intracranial ependymoma. PDGFRA, RASSF1, RB, and other relevant pathways had similar occurrence rates in both the intracranial location and the spinal cord location, suggesting that these genotype expressions remain constant no matter the location [36]. Additionally, our previous work used the three vectors targeting these pathways in a lentiviral triple cocktail in minipig spinal cord, producing high-grade glioma [21].

Our previous work showed that pQ-MCS1-IRES-eGFP retroviral overexpression of PDGF in the midthoracic spinal cord (injected midline) produced high-grade glioma in 100% of rats 4 to 7 weeks post-surgery [12]. In this study, our PDGF-B group (group 2) displayed similar results, but both progression timelines were slower than our three-vector cocktail. Tumor progression in PDGF models of glioma can be enhanced with a combination of other genetic pathways [37], and more factors are needed for tumor progression upon PDGF-B stimulation [38]. Moreover, because of glioblastoma (GBM) intratumoral heterogeneity in humans, PDGF genes display specific expression patterns through different areas of a GBM. Additionally, the expression of PDGF genes vary based on the location of the lesion within the brain [39]. Previous work with PDGF-B-driven tumors recapitulated the proneural subtype of GBM [21]. PDGF-B activating mutations are involved in angiogenesis promotion during tumor development through the recruitment of pericytes. Loss-of-function mutations of PDGF-B are associated with calcification of the brain likely due to a lack of pericyte recruitment [40]. Thus, tumor formation, progression, and makeup in the clinical setting will be highly variable, much like the PDGF-B driven models produced recently [41].

As discussed previously, HRAS is less common in the initial development of human GBM. Thus, the present study aimed to determine the need for activated HRAS in producing high-grade glioma. Rats injected with HRAS alone (group 3) or all three vectors combined (group 8) produced the most aggressive lesions. One of the most frequently observed RAS mutations occurs in the 12th amino acid, where glycine is substituted for valine (G12V). This alteration is seen in numerous cancers. While this particular RAS-mutation is not seen in glioma, the RAS-map pathway is altered in many gliomas. HRAS mutations have been shown to be involved in brain gliomagenesis and proliferation through transcriptional downregulation and activation, respectively [42,43]. Interestingly, Salirasib, a RAS inhibitor, has been shown to be an effective treatment for a rat model of GBM [44,45]. One report suggested that activated RAS in numerous human glioma cell lines can induce cell degeneration similar to that of necrosis, and different from apoptosis [46]. Our groups injected with HRAS support this theory, displaying a greater incidence of necrosis and pre-necrosis-like areas than groups without HRAS. The existing data on RAS indicate a highly complex set of roles for the RAS pathway in cancer cells.

One of the most commonly used pathways in cancer modeling is the p53 pathway. This is because p53 signaling is commonly altered in human glioma and other cancer types. This pathway responds to stress signals that influence cellular homeostatic mechanisms involving DNA replication, chromosome segregation, and cell division [47]. In the present study, p53 alone could not induce tumor formation in the given time period. Accordingly, several questions for further investigation arise. One such question in our study was whether certain groups, for example, the p53 single vector, needed a longer follow-up period to allow for lesion formation. A slower lesion growth rate could allow for a more realistic translation to human glioma growth characteristics. Future plans for evaluation include performing MRI scanning intermediately over the study period, which could be informative for lesion formation start point and growth pattern differences between groups. Moreover, we will delve deeper into genotyping our model for numerous interests, including CDKN2A deletion [48], EGFR amplification [49], IDH1/2 [50], FGFR-TACC3 [51], and H3K27M mutation [52] because of their prevalence in human glioblastoma. Optimizations such as those discussed above could allow for both a better match to the human form, as well as a better understanding of the genomic makeup for precision treatment options.

## 4. Materials and Methods

### 4.1. Vector Design

HIV-based lentiviral vectors (Vector 1: PDGF-B-IRES-eGFP, PDGF-B; Vector 2: HRAS-G12V-IRES-mPlum, HRAS; Vector 3: shRNA p53, sh-p53) were produced and stored separately to avoid the risk of transduction efficiency loss due to repeated freeze–thaws and for biosafety requirements [21]. All lentiviral vectors were titered at >10^9^ IU/mL. Controls for vectors 1–3 only included reporters and shRNA scramble.

### 4.2. Animals

All procedures were approved by the Emory University Institutional Animal Care and Use Committee (IACUC). Experiments were conducted in compliance with IACUC-approved protocols in coordination with the division of animal resources (DAR) and veterinary staff. Furthermore, 8- to 10-week-old Sprague Dawley rats (*n* = 8 per group, a total of 64 rats) were obtained from Charles River Laboratories. Rats were randomized into 8 groups (Figure 1A).

### 4.3. Surgical Approach

We selected the thoracolumbar spinal cord of the Sprague–Dawley rats to inject the lentiviral vectors. This location was chosen because tumors here can manifest hind-limb motor deficits upon lesion growth. We followed the procedure described in prior studies, adjusted to rat anatomy [21,27]. The rats were induced with 5% isoflurane gas, then maintained using 1.5–2.5% isoflurane. They were then moved to a stereotaxic platform where the surgical site was shaved and cleaned with alcohol and betadine three times each. They were given a subcutaneous injection of analgesic (buprenorphine) near the surgical site. Floating ribs were palpated and used as a landmark for the target injection site. An incision was made vertically along the spine to open the skin. A single-level laminectomy at the spinal level between thoracic and lumbar regions (T12/L1) was performed to expose the spinal cord for injection of virus. An injection pump was mounted and positioned over the injection site. A Hamilton syringe with an injection needle was loaded with 2 uL of virus and was attached to the pump. Rats that received vector cocktails were given 2uL virus, split in a 1:1(:1) ratio. The needle was positioned 1mm lateral to the midline and at a 2mm depth in order to target the white matter of the cord. The virus was injected into the target tissue at a rate of 1000 nL/min. The needle was left in the tissue for 1 min after completion of the injection to allow for any reflux to diminish (Figure 1B).

### 4.4. Animals’ Behavior, and Physical Examination

In order to ensure no deficits due to the surgical procedure, all rats were monitored to be fully ambulatory at most 30 min after removal from anesthesia inhalation. After recovery from anesthesia, any subjects showing surgical complications, such as edema or motor deficit immediately after surgery, were sacrificed and not included in the study, as per IACUC-approved protocol. All rats were monitored daily for motor deficits for the first 7 days post-surgery, and every 2 days after that using Tarlov and BBB Scoring. Scoring was determined by investigators blinded to group assignments. Each rat was placed in an enclosure measuring 1m by 0.5m and observed for 5–10 min for any motor deficits listed on the 21-point BBB scoring scale, where 21 is normal and 0 represents no movement in any of the three leg joints [22]. Tarlov scoring is a more general gauge of motor function, measuring the degree of paralysis in the hindlimbs within scores of 0–4 [53]. Tarlov scoring was determined in the same exam period based on the following descriptions: (0) no voluntary movement, (1) perceptible movement at the joint, (2) good joint mobility but the inability to stand, (3) ability to stand and walk, and (4) complete recovery [54]. Each rat was given a separate BBB score and Tarlov score for each hindlimb. Weight was monitored throughout the study as a clinical/behavioral parameter. Weights were recorded each monitored day during physical examination, both for baseline and post-operative days. Abnormal body weight changes relate to stress, nausea, loss of motor function, and more, making it an important variable to measure [55]. Post-operative behavioral and clinical examinations were performed daily for 7 days and every 2 days after surgery by investigators blinded to the vector assignments.

### 4.5. Tissue Processing

When an animal reached either a BBB Score < 9 for one hindlimb or 98-day post-surgery, whichever came first, it was euthanized using an intraperitoneal injection of Euthasol. Intracardiac perfusion was performed with 0.9% saline solution and 4% paraformaldehyde. Spinal cords were extracted and placed in 4% PFA for storage until paraffin embedding. Cords were cut into 5 mm blocks and embedded in paraffin. All tissue was sectioned on the transverse axis of the spinal cord at a thickness of 8 μm by the Emory Neuropathology Core.

### 4.6. Hematoxylin & Eosin and Immunohistochemistry

Deparaffinization through a series of xylene incubations followed by ethanol gradients in a standard procedure was executed prior to H&E staining (Sigma Aldrich, St. Louis, MO, USA). H&E slides were assessed qualitatively by a board-certified clinical neuropathologist (S.N.) blinded to group assignments through wide-field microscopy [56]. Immunohistochemical stains were performed with the Vectastain Kit and primary antibodies for PDGF-B (ab23914, ABCAM, San Diego, CA, USA), H-Ras (sc-29, Santa Cruz Biotechnology, Inc., Dallas, TX, USA), P53 (#2524, CST, USA), OLIG2 (P21954, Thermo Fisher Scientific, Waltham, MA, USA), glial fibrillary acidic protein (GFAP) (ab23914, ABCAM, Waltham, MA, USA), Ki67 (ab15580, ABCAM, Waltham, MA, USA), and sex determining region Y-box 2 (Sox2) (ab92494, ABCAM, Waltham, MA, USA) [57]. All slides were scanned in a raster pattern by the Neuropathology Core (Leica Aperio AT2 Slide Scanner, Leica Biosystems, Inc., Buffalo Grove, IL, USA). Images of the scans were taken with Aperio ImageScope software V12.3.3.

### 4.7. Data Analysis

Behavioral analysis was performed by investigators blinded to group vector assignments. Continuous and ordinal variables were summarized as appropriate using the mean, standard deviation (SD), median, and range. Statistical comparisons were conducted using a *t*-test, one-way ANOVA, or two-way ANOVA, where *p* < 0.05 was considered statistically significant (Prism Graphpad 9, San Diego, CA, USA) [21].

## 5. Conclusions

Lentiviral gene transfer of differing vector combinations produces differing high-grade glioma models in rodents. Here we tested three-vector cocktails, and most of them produced high-grade gliomas of varying intensity and cell heterogeneity. The combination of PDGF-B, HRAS, and sh-p53 vectors produces a more robust and efficient model of high-grade glioma than any other combination tested in the present study, which can lead to better preclinical testing for spinal cord glioma treatments.

## Data Availability

The data presented in this study are available on request from the corresponding author. The data are not publicly available due to ongoing studies.

## Supplementary Materials

The following are available online at https://www.mdpi.com/article/10.3390/ijms222312943/s1.
