# Supplementary material for: Lentiviral-Induced Spinal Cord Gliomas in Rat Model
Source: Int J Mol Sci. 2021 Nov 30;22(23):12943. doi: 10.3390/ijms222312943 (PMC8657985; doi:10.3390/ijms222312943)
Supplement: Supplementary file 1 [file ijms-22-12943-s001.zip › ijms-1461783-supplementary.pdf]

Figure S1

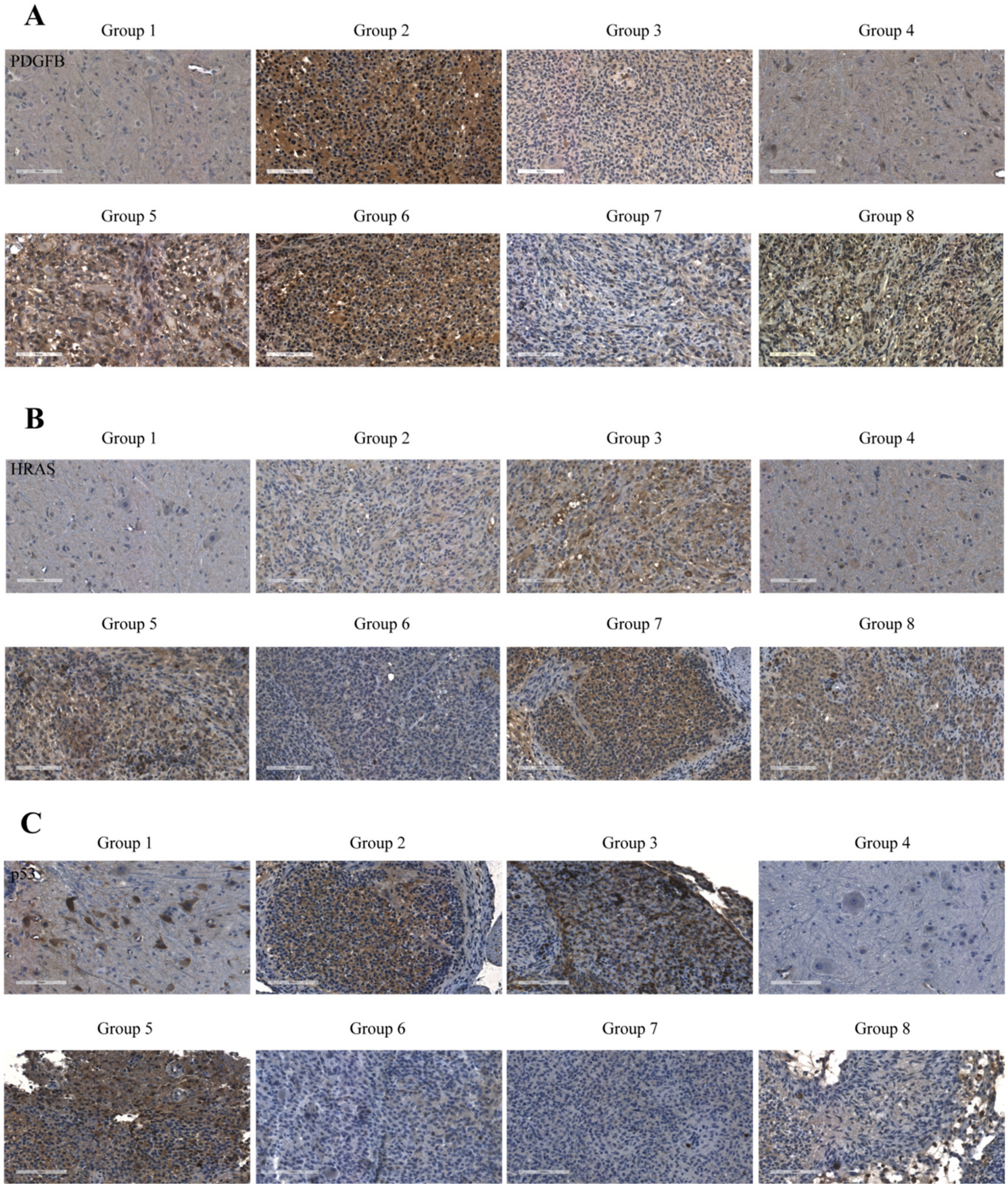

Figure S1. Immunohistochemical confirmation of lentiviral vector delivery and expression. Standard fixed, paraffin-embedded staining protocols with tris mediated antigen retrieval were applied to 8um thick serial sections. Stains for PDGF-B, HRAS, and p53 were performed to confirm that our vectors were delivered in our target tissue and appropriately expressed. **A:** PDGF-B positive staining was displayed in the four groups injected with PDGF-B (groups 2, 5, 6, 8). **B:** HRAS positivity was seen in the four groups injected with HRAS (groups 3, 5, 7, 8). **C:** P53 positive staining was displayed in the groups not injected with our p53 knockdown (groups 1, 2, 3, 5). Scale Bar = 100 um.

Figure S2

A

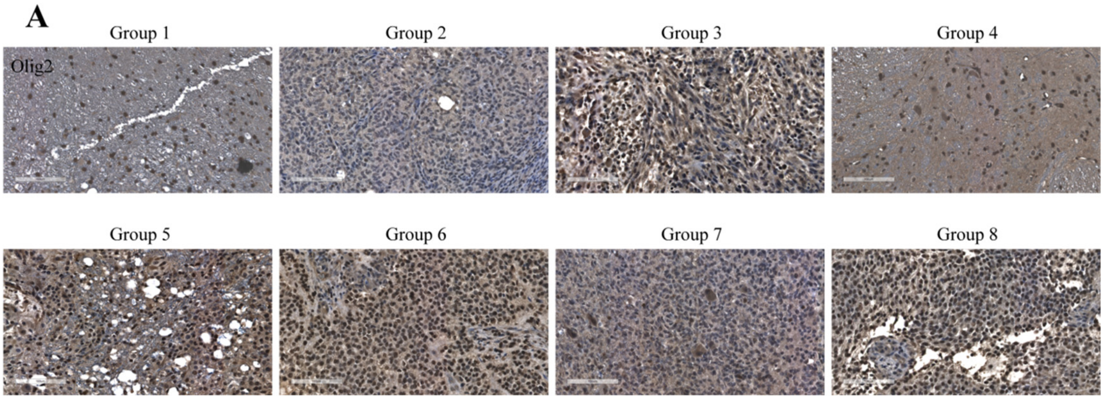

Figure S2. Immunohistochemical characterization of spinal cord lesions displaying oligodendroglial phenotype. Standard fixed, paraffin-embedded staining protocols with tris mediated antigen retrieval were applied to 8um thick serial sections. A) IHC staining for Olig2, a marker of oligodendroglial phenotype. Increased positive staining was displayed in groups injected with HRAS alone, PDGF-B+HRAS, PDGF-B+sh-p53, HRAS+sh-p53, and triple cocktail (groups 3, 5, 6, 7, 8). Scale Bar = 100 um.
